# Ancient Eastern Methods

**Published:** 1907-06-08

**Authors:** 


### The Evolution of Medicine.

### ANCIENT EASTERN METHODS.

### II.—THE MINISTRATIONS OF A HOLY MAN.

The following story is told in the "Bagh O Bahm" of a certain young prince who had fallen in love with the daughter of the King of the Jins. The young lady in question had most improperly come, uninvited and unchaperoned, to pay him a visit, and had been suddenly removed by her father. From love and longing the prince fell ill, and his father, to whom this was reported, came to visit him, bringing with him a motley crowd of healers—"acute physicians (Hakeem), truthful astrologers, wise doctors (Mullah), eunuchs, dervishes, travelling devotees (Salik), and hermits (Mujzub)." These all treated the patient simultaneously, each according to the views of the class he represented. The prince himself tells the story. "The physicians," he says, "wrote prescriptions for the defect of my brain and the strength of my heart, the doctors gave me charms and amulets to drink and to place near me. They began to pray and to blow upon me (this to exorcise demons). The astrologers said 'This appearance comes from the revolutions of the stars, be pleased to make propitiatory offerings.' In short each one said words according to his own art."

#### Appealing to a Specialist.

Some months had passed, and the patient had made no progress towards recovery, when a certain merchant arrived at the capital and was brought before the King, who inquired whether in the course of his travels he had seen or heard of "a perfect physician." The merchant replied: "Oh, Point of Adoration of the Universe, your slave has made many journeys, and in Hindoostan, in the middle of a large river there is a small hill; there a holy man of the Hindoo faith (Gusain), with matted locks, has made a temple to Shiva and an assembly house and a lovely garden. There he lives, and this is his custom. Once in the year, on the day of Shevrat, he comes out of his holy place and swims in the river and amuses himself; and after his ablutions, when he begins to go back to his deerskin praying carpet, then the sick and the possessors of pain of every country and clime, who come from a great distance, collect about his door. There is a great crowd of them. That religious man is in the habit of examining the urine and feeling the pulse, and, having written a prescription for each one, he gives it and goes away. God has given to him such a healing hand, that immediately on drinking the medicine, relief comes and the disease goes away, and remains away. I have seen this thing mine own self, and I have remembered the power of God in that he has granted such servants." The fact that both the Prince and the merchant are Mahomedans does not prevent the latter from suggesting a visit to this Hindoo idolator, nor the former from availing himself of his aid. The merchant offers to take the prince to India with him, and in support of his proposal, makes the following remark: "And it is evident that this plan is good, because, from eating the air of many countries and from change of food and water, cheerfulness may come into his temper." The virtues of a sea voyage as a cure for ill-health and depression were already recognised. The prince, the merchant, a nobleman of the state and a large retinue journey together to India. The prince thus describes the scene on the hill in the great river: "When two or three months had passed by, about four thousand sick persons assembled on that hill, and all were saying 'Now if God wishes the holy man will come out of his temple, and to all by his order there will be a complete cure. In truth, when the day came, in the morning, the holy man came out like the sun and bathed in the river and swam. Having gone across he came back again and applied the ashes of dry cow-dung to his whole body. He hid the whole of his fair body like a spark among the ashes. He made a caste mark of a superior kind of sandalwood on his forehead, fastened his loin-cloth, threw his towel over his shoulder, coiled up his topknot, twisted his mustachios, and fastened on his high shoes. From his countenance it appeared that he reckoned the whole world of no account. Having taken a jewelled pen-case under his arm he, looking in every direction and giving prescriptions, came near to me. When our four eyes met he stood still, and, pondering deeply, began to say 'Come with me.'"

#### The Open-air Treatment.

The holy man settled the prince in the lovely garden he had made, and for forty days gave him no further treatment. At the end of that period he visited him, and, being pleased with the improvement he saw in him, bade him continue to wander about the garden and eat any fruit he might fancy. He also gave him a china cup filled with a medicine compounded with honey, and bade him "Be pleased to be so good as to eat six mashahs (a mashah is about 120 grs.) without fail daily." The prince ate, and derived much benefit from the treatment, which continued on these lines for a long while. The prince beguiled the time in reading a book of magic which he had found, and not till the expiration of a year and a day did he see the holy man again.

"When the holy man came again," the prince relates, "I salaamed to him. He gave me his pen-case and said, 'Come with me.' I went with him. When he went out of the door the crowd began to bless him. The nobleman and the merchant, seeing me with him, fell at the feet of the holy man and began to give him thanks. He went to the bathing ghaut on the river according to his custom and made his ablutions and prayers. It chanced that among the crowd of lunatics his eye fell upon a young man, handsome and shapely, who from weakness had not the power to stand. He bade me bring him along. When he had given medicine and remedies to all he went into a private room."

#### An Ancient Operation.

The holy man proceeded to operate on his patient, assisted, or, at least, attended by, the prince, who thus describes the events which followed: "Having cut away a small piece of this young man's skull he desired to remove with forceps a centipede which was sitting on his brain. This came into my thought, and I up and said: 'If you make the pincers hot in the fire and put them on its back, then it is good, of itself it will come out, but if you shall pull thus, it will not let go of the pith of the brain. Then there will be fear of (the young man's) life.' Having heard this, he looked my way, and, having risen in silence, he went to a corner of the garden, and, having seized a tree in his embrace, he noosed his neck in a lock of his own long hair, and departed (this life)." Professional sensitiveness could not well go further than this. Probably the prince was the first who had been bold enough to criticise the methods of the holy man, and criticism he could not endure. The prince was very grieved at the death of his benefactor, and buried him with his own hands; then, constituting himself both his heir and his executor, he loaded all the wealth of the temple into his ships and returned home. The unhappy patient appears to have been left forgotten on the operating table.

